# Optimizing Perioperative Glycaemic Control with Continuous Glucose Monitoring in Pregestational Diabetes: Feasibility and Comparative Analysis of Two Systems: A Pilot Study

**DOI:** 10.3390/jcm14186670

**Published:** 2025-09-22

**Authors:** Joanna Kacperczyk-Bartnik, Aleksandra Urban, Paweł Bartnik, Piotr Świderczak, Aneta Malinowska-Polubiec, Aleksandra Bender, Ewa Romejko-Wolniewicz, Krzysztof Czajkowski, Jacek Sieńko

**Affiliations:** 1 II Department of Obstetrics and Gynecology, Medical University of Warsaw, 02-091 Warsaw, Poland; 2 Students’ Scientific Group, II Department of Obstetrics and Gynecology, Medical University of Warsaw, 02-091 Warsaw, Poland

**Keywords:** caesarean section, continuous glucose monitoring, feasibility study, Perioperative Glycaemic Control, pregestational diabetes mellitus

## Abstract

**Background:** Continuous glucose monitoring (CGM) has changed the clinical practice in diabetes management during pregnancy; however, its application during caesarean section remains understudied. This feasibility study evaluates the performance, reliability, and clinical utility of two CGM systems—FreeStyle Libre 2 and Medtronic Guardian Connect—during caesarean delivery and the early postpartum period in a patient with pregestational diabetes mellitus (PGDM). **Methods**: A prospective, single-patient study was conducted. A 32-year-old woman with type 1 diabetes underwent elective caesarean section at 38 weeks of gestation. Both CGM systems were applied over 18 h prior to surgery and monitored continuously through the intraoperative and five-day postpartum period. Glucose data, device performance, and usability were assessed. **Results**: Both CGM systems provided uninterrupted, high-quality glucose data throughout the perioperative period, including during spinal anaesthesia, surgical manipulation, and postoperative recovery. No sensor displacement nor signal loss occurred. Glycaemic readings remained within the normoglycaemic range (90–100 mg/dL) during surgery, with mild elevations observed during anaesthesia initiation. Postoperatively, both systems showed comparable glucose trends, with slightly lower readings from FreeStyle Libre 2. **Conclusions**: CGM is feasible and reliable during caesarean section in PGDM patients. These findings support the integration of CGM into obstetric surgical care and highlight the need for larger studies to validate clinical benefits.

## 1. Introduction

Effective glycaemic control in women with pregestational diabetes mellitus (PGDM) undergoing caesarean section is critical to minimizing perioperative risks for both the mother and the neonate. Intraoperative and postoperative hyperglycaemia have been associated with increased rates of surgical site infections, delayed wound healing, and neonatal complications such as hypoglycaemia and respiratory distress [1,2,3,4]. Standard glucose monitoring methods, typically based on intermittent capillary blood glucose measurements, may be insufficient to detect rapid glycaemic fluctuations during the perioperative period [5].

Continuous glucose monitoring (CGM) systems provide real-time data on interstitial glucose levels, offering the potential for more precise and responsive glycaemic management [6]. While CGM has demonstrated clinical benefits in the outpatient diabetes care and during pregnancy [7], its application in the surgical setting—particularly during caesarean delivery—remains under-investigated. The perioperative environment introduces unique physiological and pharmacological variables, including stress-induced hyperglycaemia, anaesthetic effects, and altered nutritional intake, which may influence both glycaemic dynamics and CGM performance [8,9]. Several CGM devices are currently available, each with distinct technological features, accuracy profiles, and usability considerations [10]. However, comparative data on their feasibility and reliability in the context of caesarean section in PGDM patients are limited. Understanding how these systems perform under surgical conditions is essential for informing clinical decision-making and optimizing perioperative glucose control.

This study aims to evaluate the feasibility of using CGM during caesarean section and the immediate postoperative period in women with pregestational diabetes. Specifically, we compare two CGM systems in terms of performance, usability, and clinical applicability in the perioperative setting: Medtronic Guardian Connect and Abbott FreeStyle Libre System.

## 2. Materials and Methods

This was a prospective, single-patient feasibility study conducted in March 2025 at the II Department of Obstetrics ang Gynecology, Medical University of Warsaw, to evaluate the performance of two continuous glucose monitoring (CGM) systems during the caesarean section and the early postpartum period. The participant was a woman with class C pregestational diabetes mellitus (type 1), aged 32, scheduled for elective caesarean delivery under spinal anaesthesia at term (38 weeks of gestation). She was receiving intensive insulin therapy via insulin pump.

Two CGM systems were used: the FreeStyle Libre 2 (Abbott Diabetes Care, Witney, UK) and the Medtronic Guardian Connect (Medtronic, Seoul, Republic of Korea). Sensors were placed on the opposite upper arms over 18 h prior to the scheduled caesarean section to allow for sensor stabilization. The Medtronic Guardian sensor was calibrated using capillary blood glucose values per manufacturer instructions, while the Freestyle Libre operated in a factory-calibrated mode.

Glucose data were collected continuously from both devices during the caesarean section and for five days postpartum. The average mean absolute relative difference (MARD) of analysed CGM devices for the operative day was calculated using temporally matched glucose data from CGM systems and comparison of two reference capillary blood glucose values [11,12]. The following mathematical formula was applied for the MARD calculation, based on self-monitored blood glucose (SMBG) values paired with CGM recorded levels:

MARD = (1/*n*) × ∑ |(CGM − SMBG)/SMBG| × 100%

The study was approved by the Medical University of Warsaw Bioethics Committee (approval number: KB/134/2024) and conducted in accordance with the Declaration of Helsinki. Written informed consent was obtained prior to participation. The project was financed from the internal Medical University of Warsaw grant (number 12/M/MB/N/24).

During the preparation of this manuscript the authors used Microsoft Copilot powered by OpenAI’s GPT-4 architecture for the purposes of grammar and spelling check of human prepared text. The authors have reviewed and edited the output and take full responsibility for the content of this publication.

## 3. Results

### 3.1. Patient’s Characteristics

The study participant was a 32-year-old woman with class C pregestational diabetes mellitus type 1, diagnosed at the age of 15. Her medical history also included hypothyroidism, managed with 50 micrograms of levothyroxine. This was her second pregnancy and second caesarean delivery. The first caesarean section was performed due to abnormal cardiotocography (CTG) findings and suspected perinatal asphyxia. In the current pregnancy, the indication for elective caesarean section at 38 weeks of gestation was severe tokophobia. Throughout the pregnancy, the patient used a Medtronic continuous glucose monitoring (CGM) system in combination with an insulin pump, with a total daily insulin dose of approximately 70 units. Insulin infusion rate was automatically controlled by glucose levels measured by Medtronic CGM feedback to the pump. The patient was familiar with functioning of both analysed systems as she used FreeStyle Libre 2 device before pregnancy. The HbA1c level at 28th gestational week was 49 mmol/mol, and increased to 62 mmol/mol in the 37th gestational week. Obtained time in range (TIR) during pregnancy was 66–72%. The caesarean section was performed under spinal anaesthesia using a Pfannenstiel skin incision and a transperitoneal approach with a low transverse uterine incision. A healthy male neonate was delivered, weighing 3795 g and measuring 53 cm in length, with Apgar scores of 10 at 1st, 3rd and 5th minutes. The neonate was classified as large for gestational age (99th percentile). The caesarean section was uncomplicated, with an estimated blood loss of 800 mL and normal postoperative wound healing.

### 3.2. Glycaemic Control During the Caesarean Section

**Figure 1:**
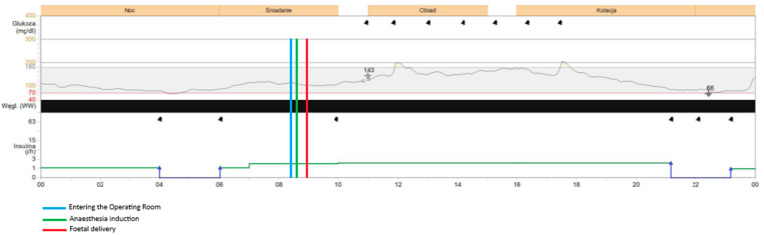
Glycaemic readings on the operative day with Medtronic Guardian Connect device.

**Figure 2:**
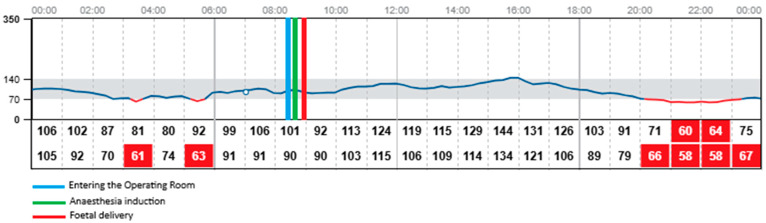
Glycaemic readings on the operative day with FreeStyle Libre 2 device. In red: results below 70 mg/dL (3.9 mmol/L).

The procedure began at 08:54 and concluded at 09:55, with the neonate delivered at 08:59. The patient was fasting since midnight. The total dosage of insulin on this day was 34 units. During the caesarean section the patient was administered insulin via the insulin pump with the dosage of 2 units per hour. Both CGM devices—FreeStyle Libre 2 and Medtronic Guardian Connect—provided continuous glucose data throughout the procedure and the early postpartum period, despite the use of bipolar electrocoagulation, repeated non-invasive blood pressure measurements, and changes in patient’s positioning required for surgical access. Continuous glucose detection by the FreeStyle Libre required the presence of a functioning mobile phone with the appropriate application installed and active within a 5-m range of the patient in the operating room. The glycaemic readings obtained from both devices were comparable throughout the monitoring period, with no clinically significant discrepancies observed. Glycaemic monitoring continued without any interruptions during the entire intraoperative phase, the patient’s transfer to the postoperative care unit, and throughout the early recovery period, with both devices remaining fully functional and providing comparable glucose readings. Both devices recorded a mild elevation in glucose levels upon the patient’s entry into the operating room (08:29) and at the initiation of the spinal anaesthesia (08:42), which may have been associated with emotional stress, anxiety, pain perception, or preoperative antibiotic administration. Both systems showed a subtle decrease in glucose levels during the surgical procedure, with values remaining stable within the normoglycaemic range of 90–100 mg/dL (5–5.55 mmol/L). Neither device registered any significant changes in glucose levels immediately before or after delivery of the neonate, nor in response to the progressive intraoperative blood loss, suggesting stable glycaemic control throughout the procedure. Figure 1. shows the results obtained during the caesarean section and in the postoperative unit with Medtronic Guardian Connect CGM. Figure 2. presents the results obtained during the same period using FreeStyle Libre 2 CGM.

### 3.3. Comparison of Obtained Results During the First 24 h Postpartum

The mean glucose concentration measured from capillary blood was 105 mg/dL (5.83 mmol/L). Glycaemic readings from Medtronic device showed mean glucose concentration of 117 mg/dL (6.5 mmol/L). FreeStyle Libre 2 measured mean glucose concentration of 95 mg/dL (5.28 mmol/L) with one episode of hypoglycaemia, 58 mg/dL (3.22 mmol/L) at 22:00, which was around 13 h postpartum. At the same time the Medtronic system measured glucose concentration of 66 mg/dL (3.67 mmol/L). The insulin dosage via pump was stopped at 21:00 as the detected glucose concentration with Medtronic device was around 80 mg/dL (4.44 mmol/L) Glucose concentration exceeding 180 mg/dL (10 mmol/L) was detected by Medtronic system altogether for 24 min on the first postoperative day and these episodes were observed during intravenous infusion of 1 g of Paracetamol (at 12:00 and 18:00). No similar observation was detected with the FreeStyle Libre 2 CGM system as the highest recorded glucose concentration on the same day was 166 mg/dL (9.22 mmol/L) measured at 16:00. None of the devices recorded changes in glycaemic trends during administration of Morphine, Ibuprofen, nor intravenous fluids. Overall glycaemic trends recorded by both devices were comparable, with slightly lower results obtained with FreeStyle Libre 2. No sensor displacement nor signal loss was observed in the postoperative period, confirming the reliability and stability of both CGM systems under clinical conditions. The patient performed two SMBG measurements of capillary blood glucose levels on the operative day. The average mean absolute relative difference (MARD) of analysed CGM devices for the operative day was below 10% for both CGM systems (Table 1).

### 3.4. Neonatal Outcomes

The neonate was born in good general condition, with Apgar scores of 10 at both 1st and 5th minutes, and remained with the mother throughout the hospitalization. There was no need for admission to the neonatal pathology unit nor the neonatal intensive care unit. Immediate skin-to-skin contact was established after birth, and breastfeeding was initiated shortly after the caesarean section finished. The infant’s birth weight was 3795 g, corresponding to the 99th percentile for gestational age. Initial glycaemic measurements in the neonate were 43 mg/dL (2.38 mmol/L) at 1 h and 15 min after birth, 46 mg/dL (2.56 mmol/L) at 2 h, and 56 mg/dL (3.11 mmol/L) at 24 h. The neonate was exclusively breastfed during the first two days of life. On the third day, a morning glucose level of 36 mg/dL (2 mmol/L) was recorded, following supplementation with formula milk, which resulted in a postprandial glucose level of 82 mg/dL (4.56 mmol/L) one hour later. Mixed feeding was continued thereafter. The neonate was discharged in good condition alongside the mother on the fifth day postpartum. Due to the maternal history of pregestational diabetes, screening ultrasound examinations of the central nervous system, abdomen, and heart were performed, all of which revealed no abnormalities.

## 4. Discussion

This feasibility study demonstrates that the use of both analysed CGM systems—FreeStyle Libre 2 and Medtronic Guardian Connect—is technically feasible and clinically reliable during the caesarean section and the early postpartum period in a patient with pregestational diabetes mellitus. Both devices provided uninterrupted, high-quality glucose data throughout the perioperative course, including during patient positioning, spinal anaesthesia, bipolar electrocoagulation use, and transport to the postoperative care unit. Importantly, no signal loss nor sensor displacement occurred, and both systems showed comparable glucose readings, supporting their utility in a surgical setting.

A mild elevation in glucose levels was observed upon entry to the operating room and during the initiation of spinal anaesthesia, likely reflecting a physiological stress response [2,9,13]. However, intraoperative glycaemia remained stable within the normoglycaemic range (90–100 mg/dL; 5–5.55 mmol/L), with no significant fluctuations associated with delivery of the neonate or blood loss. These findings suggest that CGM can provide reliable real-time data even under dynamic physiological conditions, potentially allowing for more precise insulin titration and improved glycaemic control during surgery.

To our knowledge, this is one of the first reports to directly compare two CGM systems in the intraoperative and immediate postpartum setting in a patient with type 1 diabetes. While previous studies and society recommendations have demonstrated the benefits of CGM in pregnancy, including improved neonatal outcomes and time-in-range metrics, data on its use during caesarean delivery remain limited [4,7,14,15,16,17]. Our findings support the feasibility of CGM integration into obstetric surgical care and highlight the potential for CGM to enhance perioperative glucose management in high-risk pregnancies.

While both CGM systems performed well, differences in their operational requirements were noted. The FreeStyle Libre required proximity to a functioning mobile device for continuous data capture, which may pose logistical challenges in some surgical environments [10]. In contrast, the Medtronic Guardian system, with real-time transmission and calibration features, may offer advantages in settings where uninterrupted monitoring is critical. Additionally, systems compatible with insulin pumps allow sensor-augmented pump therapy and provide potential for rapid insulin intake adjustments based on real-time glucose level recordings [8]. This option could be especially helpful for patients with more extensive or rapid glucose level fluctuations and difficulties in diabetes control [8]. In less complex clinical scenarios, the selection of a more cost-effective CGM system may be appropriate, particularly for patients with insurance coverage that includes device reimbursement. Under such conditions, depending on the location, local regulations and reimbursement policy, the monthly cost of the FreeStyle Libre 2 system may be even 10 times lower than that of the Medtronic Guardian system, representing a substantial economic advantage for both patients and payers when clinical needs allow for flexibility in device choice [10,18]. Currently, the FreeStyle Libre system is 70% reimbursed for pregnant women with diabetes in Poland, in accordance with the Regulation of the Minister of Health dated 13th October 2023, which amended the list of reimbursed medical devices issued on prescription [18].

Limitations of this study include the single-patient design. Additionally, some technical difficulties were identified during comparable analysis of the devices. It was not possible to generate and compare ambulatory glucose profiles (AGP) with time in range, time above range and time below range for the operative day (one single day) as the minimum possible time range in the LibreView platform for AGP calculation is seven days. AGP results are also missing in the detailed daily reports generated based on data from the Medtronic CGM. Another study limitation is associated with the low number of SMBG measurements used for the MARD calculations as the capillary blood glucose levels was measured twice on the operative day, which could lead to inherent statistical bias. Larger studies are needed to validate these findings and to explore whether CGM-guided insulin therapy during caesarean section can improve maternal and neonatal outcomes on a broader scale. Additionally, while both devices performed well, further comparative studies are needed to assess accuracy, usability, and integration with perioperative workflows.

## 5. Conclusions

Continuous glucose monitoring can support stable intraoperative glycaemic control and may facilitate more precise insulin management during caesarean section and postoperative period in patients with pregestational diabetes. While both analysed systems performed comparably in terms of data quality, differences in operational features and cost may influence device selection based on clinical complexity and resource availability.

## Figures and Tables

**Table 1:** The average mean absolute relative difference (MARD) of analysed CGM devices during the operative day.

SMBG	Glucose Level Measured by Medtronic CGM	Glucose Level Measure by FreeStyle Libre CGM	Medtronic CGM MARD *	Freestyle Libre CGM MARD *
7.94 mmol/L	7.67 mmol/L	6.89 mmol/L	8.5%	8.05%
3.67 mmol/L	4.17 mmol/L	3.56 mmol/L		

* The following formula was applied for MARD calculation: MARD = (1/*n*) × ∑|(CGM − SMBG)/SMBG| × 100% [11,12]. *n* = 2; SMBG—self-monitored blood glucose.

## Data Availability

Data is available from the corresponding author upon request.

## Abbreviations

The following abbreviations are used in this manuscript:

CGM	Continuous glucose monitoring
MARD	Mean absolute relative difference
SMBG	Self-monitoring of blood glucose
PGDM	Pregestational diabetes mellitus

## References

[1] Johnston R.C., Gabby L., Tith T., Eaton K., Westermann M., Wing D.A. (2017). Immediate postpartum glycemic control and risk of surgical site infection. J. Matern. Fetal Neonatal Med..

[2] Duggan E.W., Carlson K., Umpierrez G.E. (2017). Perioperative hyperglycemia management: An update. Anesthesiology.

[3] Roman A., Moreno S., Lynch T., Berghella V. (2017). 526: Intrapartum glycemic control and risk of neonatal hypoglycemia. Am. J. Obstet. Gynecol..

[4] Zhang J., Mao C., Cao Q., Huang G., Wang X. (2024). Influencing factors of glycemic control in singleton pregnancies complicated by gestational diabetes mellitus in western China: A retrospective study. Medicine.

[5] Price C.E., Fanelli J.E., Aloi J.A., Anzola S.C., Vishneski S.R., Saha A.K., Woody C.C., Segal S. (2023). Feasibility of intraoperative continuous glucose monitoring: An observational study in general surgery patients. J. Clin. Anesth..

[6] Beck R.W., Bergenstal R.M., Laffel L.M., Pickup J.C. (2019). Advances in technology for management of type 1 diabetes. Lancet.

[7] Feig D.S., Donovan L.E., Corcoy R., Murphy K.E., Amiel S.A., Hunt K.F., Asztalos E., Barrett J.F., Sanchez J.J., De Leiva A. (2017). Continuous glucose monitoring in pregnant women with type 1 diabetes (CONCEPTT): A multicentre international randomised controlled trial. Lancet.

[8] Beato-Víbora P.I., Arroyo-Díez F.J. (2018). Optimal glycaemic control during caesarean section provided by sensor-augmented pump therapy with predictive low-glucose suspend function. Acta Diabetol..

[9] Duggan E., Chen Y. (2019). Glycemic management in the operating room: Screening, monitoring, oral hypoglycemics, and insulin therapy. Curr. Diab Rep..

[10] Funtanilla V.D., Candidate P., Caliendo T., Hilas O. (2019). Continuous glucose monitoring: A review of available systems. Pharm. Ther..

[11] Freckmann G., Mende J., Pleus S., Waldenmaier D., Baumstark A., Jendrike N., Haug C. (2022). Mean Absolute Relative Difference of Blood Glucose Monitoring Systems and Relationship to ISO 15197. J. Diabetes Sci. Technol..

[12] Heinemann L., Schoemaker M., Schmelzeisen-Redecker G., Hinzmann R., Kassab A., Freckmann G., Reiterer F., Del Re L. (2020). Benefits and Limitations of MARD as a Performance Parameter for Continuous Glucose Monitoring in the Interstitial Space. J. Diabetes Sci. Technol..

[13] Dogra P., Anastasopoulou C., Jialal I. (2019). Diabetic Perioperative Management.

[14] American Diabetes Association (2020). Management of Diabetes in Pregnancy: Standards of medical care in diabetes-2020. Diabetes Care.

[15] Battelino T., Danne T., Bergenstal R.M., Amiel S.A., Beck R., Biester T., Bosi E., Buckingham B.A., Cefalu W.T., Close K.L. (2019). Clinical targets for continuous glucose monitoring data interpretation: Recommendations from the international consensus on time in range. Diabetes Care.

[16] Majewska A., Stanirowski P.J., Tatur J., Wojda B., Radosz I., Wielgos M., Bomba-Opon D.A. (2023). Flash glucose monitoring in gestational diabetes mellitus (FLAMINGO): A randomised controlled trial. Acta Diabetol..

[17] Majewska A., Stanirowski P.J., Wielgoś M., Bomba-Opoń D. (2022). Efficacy of Continuous Glucose Monitoring on Glycaemic Control in Pregnant Women with Gestational Diabetes Mellitus-A Systematic Review. J. Clin. Med..

[18] Regulation of the Polish Minister of Health from 13th October 2023. https://isap.sejm.gov.pl/isap.nsf/download.xsp/WDU20230002461/O/D20232461.pdf.

